# Use of the Vocera Communications Badge Improves Public Safety Response Times

**DOI:** 10.1155/2016/7158268

**Published:** 2016-04-04

**Authors:** Jeremy D. Joslin, David Goldberger, Loretta Johnson, D. Paul Waltz

**Affiliations:** ^1^Department of Emergency Medicine, SUNY Upstate Medical University, Syracuse, NY 13210, USA; ^2^New York State University Police Department, SUNY Upstate Medical University, Syracuse, NY 13210, USA

## Abstract

*Objectives*. Violence in the Emergency Department (ED) has been a long-standing issue complicated by deficiencies in staff training, ease of weapons access, and response availability of public safety officers. The Vocera Badge is being used by our staff to request public safety assistance in lieu of a formal phone call to the University Police Communications Center. We sought to learn if use of this technology improved officer response times to the ED.* Methods*. Mean response times were reviewed and descriptive statistics analyzed to determine if the use of the Vocera Badge improved public safety officer response times to the ED.* Results*. Average response times improved from an average of 3.2 minutes (SD = 0.456) in the 6 months before the use of the communication badges to an average of 1.02 minutes (SD = 0.319) in the 6 months after use began.* Conclusions*. The use of the Vocera Badge seemed to decrease response times of public safety officers to our ED compared with the traditional method of calling a dispatch center to request assistance.

## 1. Introduction

Violence in the Emergency Department (ED) has been a long-standing issue with much recognition reported in the medical literature. Previously cited contributing factors to ED violence include deficiencies in ED staffing and training; ease of access of weapons in the ED; and the lack of helpfulness, lack of presence, and delayed response times of public safety officers [1]. Indeed, firearms, knives, chemical sprays, and other weapons have all been retrieved by metal detectors installed at ED entrances according to a recent report [2].

Behnam et al. found that 78% of EDs surveyed reported at least one violent act in the previous 12 months and that workplace violence in the ED was more common in those EDs with annual volumes over 60,000 patients [3]. Indicators of potential violence and difficult behaviors are being identified [4] which will hopefully lead to timelier request for public safety or law enforcement personnel presence. Nursing and other staff identification of what constitutes violence may be taken for granted as “just part of my job” resulting in underreporting [5].

The Vocera Badge (Vocera Communications, San Jose, CA) is a Wi-Fi networked, hands-free communication device used amongst our ED staff for real-time voice communications. All attending physicians, resident physicians, nurses, clerical staff, and housekeeping staff use the device as part of their routine communication workflows. Use of this device has been shown to reduce total distances walked by inpatient ward nurses during a shift, thereby increasing communication effectiveness and efficiency amongst staff [6]. In an academic ED setting, use of the device has been shown to decrease the number of interruptions and the duration of those interruptions [7].

The Upstate Medical Center (University Hospital Campus) Emergency Department is an urban, level 1 trauma and tertiary care center with an approximate annual volume of 73,000 patient visits (2014) located in Syracuse, NY. Upstate Medical University is part of the State University of New York system and enjoys the presence of the New York State University Police Department, a full-time, campus law enforcement agency that includes a public safety division providing additional coverage for the hospital and ED. Requests for assistance are answered by a tiered approach with either public safety officers responding, police officers responding, or both depending on the nature of the request. Public safety officers are mostly stationed within the hospital and around campus, whereas police officers patrol and interact within the hospital and our multiple city block campus.

Around July 1, 2014, the University Police Department outfitted three public safety personnel with Vocera Badges. All ED staff had already been using these badges for day-to-day communication needs. In the event of a violent threat, any ED staff member could summon the assistance of police and public safety using their Vocera Badge. Calls for assistance could still be placed in the tradition manner as well.

Previous to this, a telephone call to the dispatch center would need to be placed where questions such as “What is the nature of your call?” and “Where are you located?” had to be answered. After the completion of this call an officer could then be dispatched.

Review of response times to the ED for “calls for assistance” was undergone to determine if this intervention decreased response times and effectively improved perceived safety with the potential goal of reducing violence within our ED.

## 2. Methods

We performed a retrospective review of University Police Department call logs after our institution's review board determined the project to be “not human research” and therefore did not require full IRB review (Project #733298-1).

Due to internal information security policies, only anonymized and summarized data was made available from the University Police Department's computer assisted dispatch (CAD) system using ARMS (version 3 R3). Both the total number and the average (mean) response times for all responses to the ED during each month between January 2014 and December 2014 were exported. Individual response times were not made available for review.

Comparison of response times between preimplementation months (January through June) and postimplementation months (July through December) was made, as well as other descriptive statistics. Response time was defined as the time from placing a call for assistance to the time of arrival of the first officer on the scene (either public safety officer or commissioned police office). Limited descriptive analysis was performed including an independent samples *t*-test that was conducted on the mean response times for the 6 months before use of the communication badges and the 6 months after use began.

## 3. Results

A total of 3,606 calls for assistance were made during the study period with a call volume range of 230 calls during the month of September to 387 calls during the month of January. The mean call volume per month before implementation was 327 calls and after implementation was 274 calls.  Table 1 shows the volume of calls and the mean response times for calls by month.

The mean response time for calls during the preimplementation months was 3.2 minutes (SD = 0.456) with a range of 2.8 minutes in the month of June to 3.8 minutes in the month of January. The mean response time for calls during the postimplementation months was 1.02 minutes (SD = 0.319) with a range of 0.7 minutes in September to 1.5 minutes in the month of August, *t*(10) = 9.61, *p* < 0.001.

## 4. Discussion

**Figure 1:**
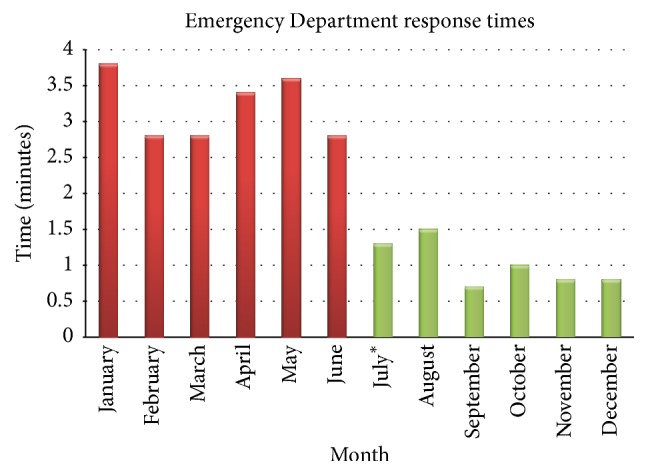
Mean response times of police and public safety officers to our ED by month.

Our data seems to be the first report describing the use of a hands-free communication device such as the Vocera Badge to improve public safety response times and potentially decrease violence in the ED. Though our analysis is somewhat limited due to information security policies, it appears that the use of the Vocera Badge to request assistance to the ED seems to have dramatically reduced response times of police and public safety officers at our hospital.  Figure 1 graphically depicts this reduction after intervention.

From the time of implementation in July, response times fell significantly. The reduced response times remained consistently lower than before implementation throughout the entire study period. Though response times were collected from two different seasons, we do not feel that seasons or weather affected results since the first arriving public safety officer is likely to have responded from within the hospital. This reduced response time was subjectively felt by ED staff. Unpublished survey results obtained from our ED staff after implementation revealed an increased sense of safety because of this.

## 5. Limitations

Our data may be affected by a few limitations. Firstly, this is a single sample location. It is unknown if this effect is reproducible at other facilities. Other factors could have been involved with improving the response times. Of note, a new University Police Department's chief of police (DPW) took office around this time. It is possible that his presence and renewed efforts to improve safety had an impact as well as the intervention studied. Indeed, one additional initiative involving the concept of community policing was begun around this time and may have also contributed to our observations. The community policing initiative increased the overall presence of officers with more routine “rounding” and patrolling. The number of public safety personnel on duty did not change during this time period.

Additional limitations include the possibility of reporting selection bias. That is, we were able to describe the metrics of all calls that occurred, but it is possible that some ED events did not generate a call that should have occurred. Though the use of the Vocera Badge was established as a primary means of requesting public safety assistance, it is likely that some requests were still made by telephone. Our data is a heterogeneous mixture of Vocera Badge-initiated requests and telephone-initiated requests.

Ideally, we would have been able to analyze data on a per-call basis instead of pooled data—a requirement of our institution's security policies. Though the distinction is subtle, our null hypothesis needed to be that average monthly response times were unaffected by implementation of the Vocera device instead of the more “pure” null hypothesis we would have preferred: that response times for individual calls were not different after implementation. Finally, because of the intervention, the results are not blinded. It is possible that a Hawthorne effect is what improved the response times.

## 6. Conclusions

The use of the Vocera Badge seemed to decrease response times of public safety officers to our ED compared with the traditional method of calling a dispatch center to request assistance. It is possible that these improved response times decreased potential violence occurring in the ED.

## Figures and Tables

**Table 1:** Calls for assistance to the Emergency Department by month.

	Call volume	Mean response time (minutes)
January	387	3.8
February	304	2.8
March	309	2.8
April	329	3.4
May	350	3.6
June	283	2.8
July	316	1.3
August	337	1.5
September	230	0.7
October	264	1
November	244	0.8
December	253	0.8
